# Rapid selection and identification of *Miscanthus* genotypes with enhanced glucan and xylan yields from hydrothermal pretreatment followed by enzymatic hydrolysis

**DOI:** 10.1186/1754-6834-5-56

**Published:** 2012-08-03

**Authors:** Taiying Zhang, Charles E Wyman, Katrin Jakob, Bin Yang

**Affiliations:** 1 Center for Environmental Research and Technology, Bourns College of Engineering, University of California, 1084 Columbia Avenue, Riverside, CA, 92507, USA; 2 Chemical and Environmental Engineering Department, Bourns College of Engineering, University of California, Riverside, CA, 92521, USA; 3 Mendel Biotechnology Inc., 3935 Point Eden Way, Hayward, CA, 94545, USA; 4 Center for Bioproducts and Bioenergy, Washington State University, 2710 University Drive, Richland, WA, 99354, USA

**Keywords:** *Miscanthus*, High throughput, Hydrothermal pretreatment, Co-hydrolysis

## Abstract

**Background:**

Because many *Miscanthus* genotypes can be cultivated with relatively high productivity and carbohydrate content, *Miscanthus* has great potential as an energy crop that can support large scale biological production of biofuels.

**Results:**

In this study, batch hydrothermal pretreatment at 180°C for 35 min followed by enzymatic hydrolysis was shown to give the highest total sugar yields for *Miscanthus x giganteus cv. Illinois* planted in Illinois. High throughput pretreatment at 180°C for 35 min and 17.5 min followed by co-hydrolysis in a multi-well batch reactor identified two varieties out of 80 that had significantly higher sugar yields from pretreatment and enzymatic hydrolysis than others. The differences in performance were then related to compositions of the 80 varieties to provide insights into desirable traits for *Miscanthus* that enhance sugar yields.

**Conclusions:**

High throughput pretreatment and co-hydrolysis (HTPH) rapidly identified promising genotypes from a wide range of *Miscanthus* genotypes, including hybrids of *Miscanthus sacchariflorus/M. sinensis* and *Miscanthus lutarioriparius,* differentiating the more commercially promising species from the rest. The total glucan plus xylan content in *Miscanthus* appeared to influence both mass and theoretical yields, while lignin and ash contents did not have a predictable influence on performance.

## Background

World petroleum reserves are being depleted at an accelerating rate and production rates are slowing. In fact, Kerr reported that conventional oil production might have already reached its peak instead of plateauing between 2015 and 2020 as predicted earlier [1-3]. In addition, a move from fossil to renewable fuels is vital if we hope to slow the impacts of carbon dioxide accumulation on global climate. Against this background, production of fuels from cellulosic biomass is one promising option for large-scale and low-cost sustainable production of liquid fuels with low greenhouse gas emissions. Crops planted for the specific purpose of energy production are expected to play an essential role in meeting the Energy Independence and Security Act of 2007 in the United States. In the recent report entitled “U. S. Billion Ton update: Biomass Supply for Bioenergy and Bioproducts Industry” jointly released by DOE and USDA, energy crops were predicted to become dominant at prices above $50 per dry ton after 2022 with the baseline about 37% of total biomass at $60 per dry total [4]. Among various potential perennial energy crops, *Miscanthus*, which is native to Asia and the Pacific Islands, has been selected and studied as one of the most promising energy crops for Europe over the past two decades [5-8]. Based on *Miscanthus* field trials at different locations in Europe, a growth model towards more robust yield predication on the basis of different climatic and soil condition was developed in Europe [9]. Since *Miscanthus* has been successfully produced with high yields over a wide range of climatic conditions in Europe, it also became of interest as a dedicated biomass crop in the US, and first field trials were planted in Illinois and Arkansas [10-14]. It has been reported that the average productivity of *Miscanthus* x giganteus was 30 t/ha-year and the maximum productivity was as high as 61 t/ha-year over a 3 year period in side-by-side trials while the productivity of switchgrass, one of the most studied energy crops in the United States, was reported to be 15.8 t/ha-year in upland and 12.6 t/ha-year in lowland varieties [10]. Thus, *Miscanthus* could potentially reduce the land requirements to support U.S. biofuels production [11,12]. Further study on the effects of management on *Miscanthus* x *giganteus* productivity in different environments based on four locations in the United States provided more insights on how to improve the productivity of *Miscanthus* x *giganteus* and its capacity as a stable and reliable biomass feedstock [13]. In addition to high productivity, *Miscanthus* could be very important for the relatively high carbohydrate content (>60%) of some genotypes. The *Miscanthus* genus in general, and *Miscanthus* x *giganteus* in particular, have been identified as prime candidates for biomass energy crops because of an array of other attributes including high photosynthetic efficiency, strong stress tolerance, perennial growth, low nutrient requirements, and high carbon content [5,14]. For biological processing to ethanol and other products, it is particularly beneficial to identify genotypes that are most easily processed into sugars.

As with other types of lignocellulosic biomass, pretreatment is essential to disrupt the natural recalcitrance of *Miscanthus* to release fermentable sugars with high yields with the fungal enzymes typically used. However, as summarized in Table 1, only a few studies have been published to determine the performance of different pretreatment methods followed by enzymatic hydrolysis for conversion of *Miscanthus* into fermentable sugars, including mechanical size reduction with sodium chlorite delignification [15], ammonia fiber expansion (AFEX) [16], one step extrusion/NaOH pretreatment [14], ethanol organosolv processing [17], dilute acid steam explosion [18], and treatment with alkaline peroxide combined with electrolyzed water [19]. Overall, sugar yields from cellulose and hemicellulose, as a percent of the maximum possible, were reported to vary from 61.3% [18] to 98% [17] and from 38% [14] to 100% [15], respectively. 

**Table 1:** **Summary of *Miscanthus*pretreatment data reported in the literature **

**Pretreatment**		Overall conversion*, %		**Ref.**
		**Glucan**	**Xylan**	
ball-milling: NaCl/glacial acetic acid, 70°C, 1 h		100	90.6	[15]
ammonia fiber expansion: 60°C, 5 min		96	81	[16]
one step extrusion/NaOH: solid to liquid ratio of 1:6, NaOH 12%(w/w) pretreatment at 70°C for 4 h		69	38	[14]
ethanol organosolv process		98	73	[17]
diluted acid explosion: 0.75% H_2_SO_4_ at 100°C for 14 h; atmospheric air/H_2_O_2_ wet explosion with solid loading 15% at 170°C under 200 bar for 5 min	air	61.3	94.9	[18]
	H_2_O_2_	63.7	82.4	
alkaline peroxide: (50°C, 24 h), electrolyzed water at 121°C for 50 min		84	N/A	[19]

* Overall conversion refers to total conversion resulted from pretreatment and sequential enzymatic hydrolysis on the basis of original glucan or xylan content of raw *Miscanthus*.

Besides being affected by pretreatment methods, the fermentable sugar yields of *Miscanthus* were strongly influenced by the genotype, production site, climate, age, and plant part(s) harvested. Biomass quality was also impacted by such factors as cellulose and lignin biosynthesis and deposition and extractives [20,21]. New functional genomics and plant biotechnology tools could genetically optimize *Miscanthus* for liquid fuel production by identification of genes that improve breakdown to sugars through modification of growth rates, environmental stress tolerance, and cell wall composition, as being applied to switchgrass and alfalfa [22,23]. However, because the effect of changes in plant structure on sugar release cannot yet be predicted a’ priori, pretreatment and enzymatic hydrolysis must currently be directly applied to evaluate how sugar release changes with genetic modifications in biomass so we can identify traits that are desirable for biofuels feedstocks.

Among pretreatment options, hydrothermal pretreatment with just hot water has produced reasonably high sugar yields with various biomass feedstocks, such as corn stover [24], wheat straw [25], switchgrass [26], and poplar [27]. Furthermore, hydrothermal pretreatment advantages include no requirement for chemical additions, simple operation, and low cost materials of construction that would be of great economic advantage if high total sugar yields could be realized. Thus, it is highly desirable to indentify *Miscanthus* plants that achieve high sugar yields from the coupled operations of hydrothermal pretreatment and enzymatic hydrolysis.

Conventional pretreatment and enzymatic hydrolysis methods are very labor intensive and time consuming, making it very expensive and/or slow to screen large numbers of plants to find those that display enhanced sugar yields. However, high throughput methods have been recently developed that allow rapid screening of large numbers of combinations of plants, pretreatment conditions, and enzyme loadings and formulations to narrow the field to those with high sugar release or other desirable features [28-34]. These methods can also handle much smaller amounts of samples than conventional approaches, thereby allowing characterization of sugar release from different anatomical fractions [30]. Such rapid screening methods have been applied to a sorghum diversity panel [32], a high-throughput microplate for enzymatic hydrolysis of lignocellulosic biomass [33], and high-throughput screening of cellulose following ionic liquid treatment [34]. Recent rapid screening studies of enzymatic hydrolysis using different glycosyl hydrolases were employed to compare AFEX and dilute acid pretreatment of corn stover [35,36]. Studer et al. developed a higher temperature, high throughput method appropriate for hydrothermal and other thermochemical pretreatments based on the 96 well-plate format and employing a custom made steam chamber for rapid heating and cool down of multiple reaction vessels [29]. Results for poplar without liquid–solid separation after pretreatment in the multi-well plate system were shown to be statistically identical to those from standard pretreatment and hydrolysis methods with liquid–solid separation and solid washing [29]. Advantage has also been taken of the ability to process small sample sizes to determine variations in sugar release among tree growth rings and to identify promising traits in poplar as well as the high throughput determination of glucan and xylan fractions in lignocelluloses developed by Selig et al. [28,31,37].

In this study, the previously established HTPH system was applied to screen *Miscanthus* genotypes with the goal of identifying those that displayed enhanced release of glucan and xylan from the coupled operations of hydrothermal pretreatment and enzymatic hydrolysis. Conventional hydrothermal pretreatment in tubular batch reactors followed by enzymatic hydrolysis was applied first to identify baseline conditions that gave the highest total sugar yields from *Miscanthusx giganteus cv. Illinois* planted in Illinois. Then the HTPH system was applied to 80 different *Miscanthus* varieties to screen for those that gave the highest sugar release and identify the most promising genotypes based on baseline conditions determined from conventional pretreatment results. As reported in a companion paper (in preparation), a flowthrough reactor system was then applied to follow release of sugars and other biomass components and gain new insights into biomass deconstruction patterns that favour high sugar yields based on hydrothermal pretreatment.

## Results and discussion

### *Miscanthus* compositions

As the most widely cultivated biomass genotype currently available, *Miscanthus x giganteus cv. Illinois* was selected as the reference material for identification of pretreatment conditions that gave the highest total glucose plus xylose release from conventional pretreatment and subsequent enzymatic hydrolysis in the batch tubes. This genotype contained 42.87 ± 0.64% glucan, 22.02 ± 0.32% xylan, 19.67 ± 0.01% lignin, 2.33 ± 0.10% ash, 3.21% water extractives, and 5.80% ethanol extractives. The average moisture content of *Miscanthus x giganteus cv. Illinois* was determined to be 7.37 ± 0.11% based on 10 tests. The data reported for the 80 *Miscanthus* genotypes (provided as the Additional file 1: Table S1 and Additional file 2: Table S2) include averages, minimum and maximum contents of glucan, xylan, the total glucan plus xylan as carbohydrates, and lignin. The average compositions of all 80 *Miscanthus* genotypes were 40.74% glucan, 21.01% xylan, 24.03% lignin, and 2.83% ash. However, the 80 *Miscanthus* genotypes showed significant diversity in compositions, with glucan levels ranging from 27.7% to 48.6%, xylan from 19.6% to 27.1%, lignin from 15.5% to 27.8%, and ash from 1.10% to 7.37%. Thus, these samples were expected to enable the study of how composition affects sugar release from pretreatment and hydrolysis and aid in the selection of desirable traits to target for improvements. The total glucan and xylan content, which was important to establish the maximum possible ethanol yield, ranged from 48.6% to 72.8%. The genotypes with the highest total glucan and xylan content of 48.6% glucan and 24.2% xylan also had the highest glucan content and could realize a theoretical ethanol yield of 127 gallons from glucan and xylan per dry ton of feedstock, as calculated by the DOE Theoretical Ethanol Yield Calculator [38]. The theoretical ethanol yield of *Miscanthus x giganteus cv. Illinois* was 113 gallons ethanol per dry ton feedstock. Therefore, *Miscanthus* has excellent potential for high ethanol yields.

### Batch pretreatment and enzymatic hydrolysis

**Figure 1:**
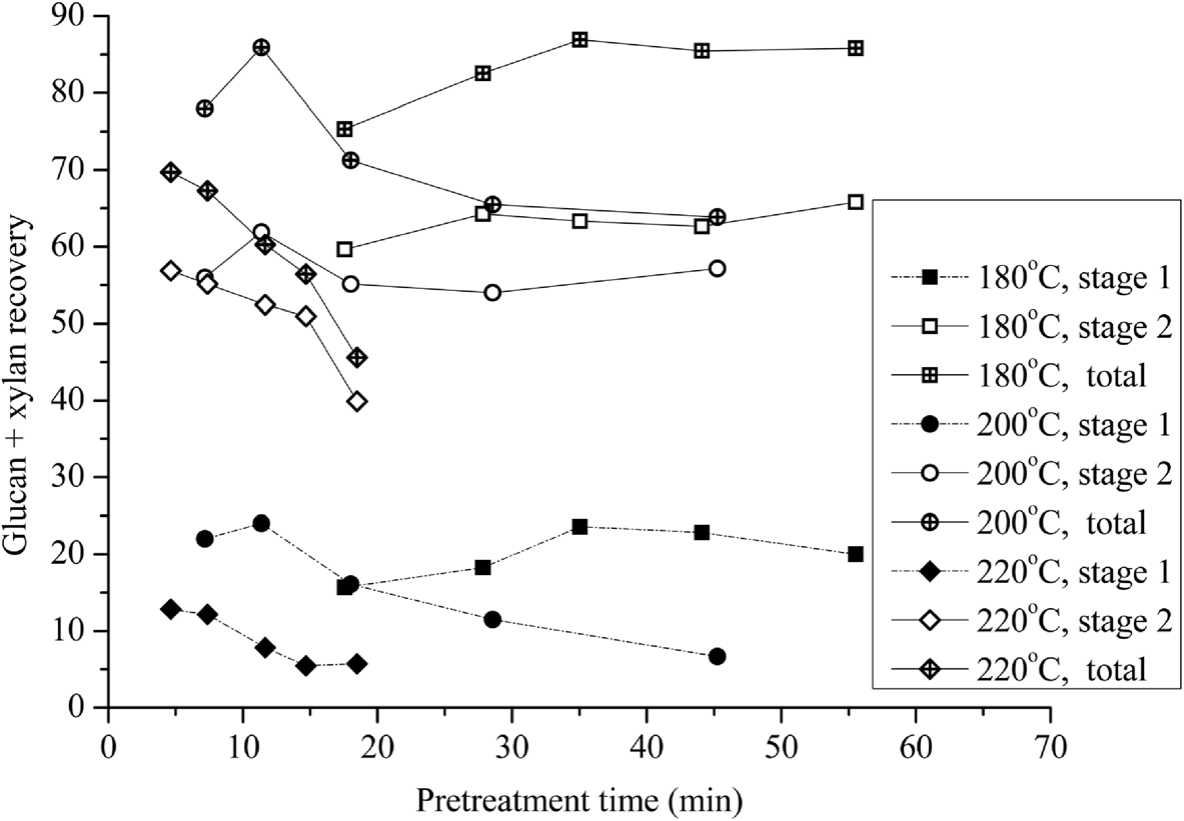
**Glucan plus xylan yields vs. pretreatment time from hydrothermal pretreatment of *Miscanthus x giganteus cv. Illinois.*** Hydrothermal pretreatments at 180°C, 200°C and 220°C followed by enzymatic hydrolysis with 60 FPU/g(glucan + xylan) at 50°C for 72 hours.

Figure 1 summarizes glucan and xylan sugar yields from batch hydrothermal pretreatment (Stage 1) of *Miscanthus x giganteus cv. Illinois* in tube reactors at 180°C, 200°C, and 220°C and from subsequent enzymatic hydrolysis of the washed solids (Stage 2) at the conditions noted. Soluble xylan and glucan yields in Stage 1 increased with pretreatment time at 180°C and 200°C to peak values after 35 and 11.4 minutes, respectively, before dropping with longer times due to xylose degradation becoming more rapid than xylan hydrolysis to xylose. However, the glucan plus xylan based sugar yields in Stages 1 and 2 combined dropped from 69.7% to 45.6% as reaction time increased from 4.6 min to 18.5 min at 220°C because xylan degradation became very rapid and pronounced in Stage 1.

For Stage 2, glucan plus xylan yields reached peak values of 64.2 and 61.9% after 28 and 11.4 minutes for pretreatment at 180°C and 200°C, respectively, but sugar yields continually dropped with increasing pretreatment times at 220°C for the range of times tested. Sugar yields from enzymatic hydrolysis of pretreated solids were relatively high, ranging from 73.5 to 93.1% over the range of pretreatment temperatures tested.

Total glucan plus xylan yields from pretreatment (Stage 1) at 180°C combined with enzymatic hydrolysis (Stage 2) increased from 79.5% to 86.9% as pretreatment time was increased from 17.5 min to 35 min and then dropped slowly with extended pretreatment time. At 200°C, overall glucan plus xylan yields from Stage 1 plus Stage 2 increased from 78.0% to 85.9% as pretreatment time increased from 7.2 min to 11.4 min and then dropped. Increasing the temperature to 220°C resulted in a maximum overall glucan plus xylan yield of 69.7% from the two stages combined at the shortest sampling time of 4.6 min with a rapid drop after that, suggesting that the rapid degradation of xylose resulted in overall sugar loss at 220°C. Thus, similar high total sugar yields of about 85% were obtained at 180°C and 200°C for *Miscanthus x giganteus cv. Illinois* pretreatment followed by enzymatic hydrolysis, while sampling times were not short enough to capture high sugar yields at 220°C. Because such short times would be impractical to implement commercially, additional experiments were not run to determine if yields would improve at shorter times. Thus, pretreatment times to achieve the highest overall glucan plus xylan yields from the two stages for *Miscanthus x giganteus cv. Illinois* were 35 min at 180°C and 11.4 min at 200°C, corresponding to similar pretreatment severities (logR_0_) [39] of 3.9 and 4.0, respectively.

The maximum glucan plus xylan yields for hydrothermal pretreatment followed by enzymatic hydrolysis were very similar to those reported in the literature for AFEX and ethanol organosolv pretreatments of *Miscanthus,* as shown in Table 1. In addition, as a result of the higher carbohydrate content of *Miscanthus* compared to other biomass listed in Table 1, the overall mass sugar yield of 55.7 g glucan plus xylan per 100 g of dry *Miscanthus x giganteus cv. Illinois* was greater than mass yields from application of hydrothermal pretreatment to other feedstocks listed in Table 2, such as 40.8 g glucan + xylan per 100 g dry corn stover [24] and 52.6 g glucan + xylan per 100 g dry wheat straw [26]. Although pretreatment temperatures and solids loadings were different for these five feedstocks, the log of the pretreatment severities at the highest overall glucan and xylan yields were in the range from about 3.9 to 4.0 for *Miscanthus x giganteus cv. Illinois*, corn stover, and poplar. However, both corn stover and poplar studies used higher solids loadings and achieved higher xylan yields but lower glucan yields [24,40]. This suggests that corn stover and poplar require more severe hydrothermal pretreatment to disrupt the cellulose structure enough to gain higher glucan yields in Stage 2. Wheat straw was reported to require more severe pretreatment (logR_0_ of 4.15) to achieve the highest overall glucan and xylan yield, while the highest glucan yield of 91% (mainly in Stage 2) was obtained with relatively low enzyme loading of 15 FPU Celluclast plus 15 IU Novozym 188 per gram dry substrate (about 39.7 FPU cellulase per gram glucan) [25]. 

**Table 2:** **Comparison of *Miscanthus*and other selected biomass feedstocks **

**Biomass**	**Composition (% dry weight raw)**		Theoretical EtOH yield (gal/dry ton feedstock)*	**Pretreatment condition**	**Enzymatic hydrolysis**	Sugar yield **		
	**Glu**	**Xyl**				**Glu**	**Xyl**	**Total**
*Miscanthus x giganteus cv. Illinois* in this study	42.9	22.0	113	180°C, 10% solid loading, 35 min, logR_0_ 3.90	60 FPU Spezyme CP plus 120 CBU Novozym 188 per gram glucan + xylan, 72 h	92	74	55.7
Corn stover [24]	36.1	21.4	100.3	210°C, 50% solid loading, 6 min, logR_0_ 4.02	60 FPU Spezyme CP plus 120 CBU Novozym 188 per gram glucan + xylan, 72 h	61.1	87.6	40.8
Wheat straw [25]	37.8	22.8	105.6	188°C, 10% solid loading, 40 min, logR_0_ 4.19	15 FPU celluclast plus 15 IU Novozym 188 per gram dry substrate, 72 h	91	80	52.6
Switchgrass [26]	32.2	20.3	91.5	190°C, 5% solid loading, 10 min, logR_0_ 3.64	15 FPU cellulase per gram glucan, 72 h	N/A	73.1	N/A
Hybrid Poplar [27]	44	15	102.5	200°C, 15% solid loading, 10 min, logR_0_ 3.94	40 FPU Spezyme CP plus 40 CBU Novozym 188 per gram glucan, 48 h	63.3	85.3	45.5

Glu-glucan, xyl-xylan, lig-lignin.

*Theoretical EtOH yields were calculated by NREL Theoretical Ethanol Yield Calculator [38].

** Sugar yields for glu and xyl were %, the totals of glu and xyl were g/100 dry weight.

### High throughput pretreatment and co-hydrolysis (HTPH) of *Miscanthus*

**Figure 2:**
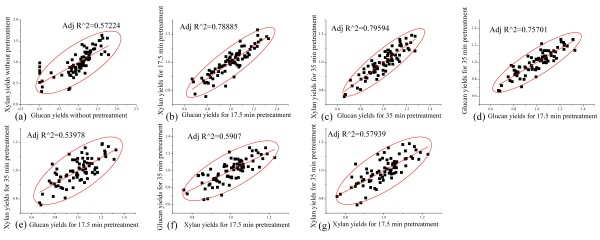
**Normalized scatter matrix of 80 *Miscanthus*HTPH glucan and xylan mass yields.** HTPH glucan and xylan mass yields (g/100 g dw raw *Miscanthus*) from hydrothermal pretreatment at 180°C in 0, 17.5 min, and 35 min.

The HTPH system was applied to 80 *Miscanthus* genotypes with different characteristics to rapidly measure overall glucan and xylan yields from hydrothermal pretreatment at 180°C for 0, 17.5, and 35 min followed by enzymatic hydrolysis of the entire pretreated slurry (180°C for 35 min is the optimal condition based on our previous batch tube experiments, data unpublished). A scatter matrix S(x) of compositions and HTPH sugar yields (mass yields, g/100 g raw *Miscanthus*) for the 80 *Miscanthus* genotypes is presented in Additional file 3: Figure S1 (selected data with R^2 > 0.5 are shown in Figure 2) X = (x_1_*x*_2_ x_10_)’ to provide a statistical estimate of the covariance matrix of the multivariate normal distribution and allow determination of whether the variables are correlated and whether the correlation is positive or negative. The data in Figure 2 and Additional file 3: Figure S1 were normalized by the average values of 80 *Miscanthus* genotypes. The 10 components in the scatter matrix (4 compositional variables plus 6 sugar yields for three different pretreatment times followed by co-enzymatic hydrolysis) were assumed independent so that the regular covariance matrix would be a diagonal matrix. The diagonal in Additional file 3: Figure S1 was filled with the variables (x_i_) of the scatter matrix for each column, and the results and discussion focuses on the lower left part in Additional file 3: Figure S1. Additional file 3: Figure S1 included all the scatter plots with ellipse matrix (x_i_’) and linear fit applied at the 95% confidence level with the adjusted R-square between any of ten variables in order to reveal correlations. For example, the first column (x_1_) (K-lignin column) showed the scatter plots of other nine variables (x_i,_ i = 2-9) as listed in each row in the diagonal versus K-lignin content with ellipse matrix and linear fit with the adjusted R-square to investigate the correlations. No obvious correlation was found between sugar yields from enzymatic hydrolysis of raw *Miscanthus* and lignin contents, but a negative correlation was observed between sugar yields and lignin contents following hydrothermal pretreatment for 17.5 and 35 min, consistent with recently reported findings for poplar [28]. 

In the second left column (glucan content column) in Additional file 3: Figure S1, a positive correlation was found between glucan and xylan contents (the third row) in raw *Miscanthus*, suggesting a structural correlation between them. A negative correlation was observed between glucan contents and sugar yields for enzymatic hydrolysis without pretreatment, suggesting cellulose in raw biomass was a strong contributor to recalcitrance [15,19,25]. However, overall mass sugar yields (g sugar/100 g biomass) following pretreatments at 17.5 and 35 min in the HTPH system were positively correlated to glucan content, which simply emphasized that high glucan content was one of the vital criteria for cellulosic feedstocks.

Correlations between xylan content and other compositions and sugar yields (the third left column (xylan content column) in Additional file 3: Figure S1) were similar to correlations found for glucan content, except that sugar yields from enzymatic hydrolysis of raw *Miscanthus* were less negatively correlated to xylan content, undoubtedly due to the lower recalcitrance of xylan than glucan.

Correlations between ash content and other compositions and sugar yields are shown in the fourth left column (ash content column) in Additional file 3: Figure S1. Although a negative correlation was found between K-lignin content and ash content in raw *Miscanthus*, glucan content and xylan content were not obviously related to ash content. This indicated that the small amount of ash did not appear to affect structural carbohydrates content. Sugar yields did not show any significant correlation to ash content for pretreatment times of 0, 17.5, and 35 min followed by enzymatic hydrolysis.

Besides the correlations of the composition variables and sugar yields, glucan and xylan yields for three different pretreatment times showed interesting and most significant correlations (Figure 2). The strongest positive correlations were found between HTPH glucan and xylan yields with R^2 values of 0.5772, 0.7888, and 0.7959 for the pretreatments at 0 min, 17.5 min and 35 min, respectively (Figures 2a, 2b, and 2c). These results suggested a strong correlation of glucan and xylan yield for the HTPH system and followed the sugar yield patterns observed for application of HTPH to poplar [28]. However, the glucan or xylan yields without pretreatment did not show correlations (with R^2 less to 0.05) to those glucan and xylan yields for the pretreatment at 17.5 min and 35 min ( Additional file 3: Figure S1). These results suggested that sugar yields from direct enzymatic hydrolysis of raw lignocellulosic biomass without pretreatment were not suitable predictors of sugar yields from enzymatic hydrolysis of pretreated biomass. However, the glucan yields for pretreatment at 17.5 min showed a very strong positive correlation (with R^2 of 0.757) to glucan yields for pretreatment at 35 min (Figure 2d). The correlation (with R^2 of 0.5907) between xylan yields for 17.5 min pretreatment and glucan yields for 35 min pretreatment (Figure 2f) was not as strong as that between glucan yields (Figure 2d) although positive correlations were found between xylan yields for 17.5 min pretreatment and glucan for 35 min pretreatment (R^2 of 0.5907, Figure 2f), and between xylan yields for 17.5 min and for 35 min pretreatment (R^2 of 0.5794, Figure 2g).

**Figure 3:**
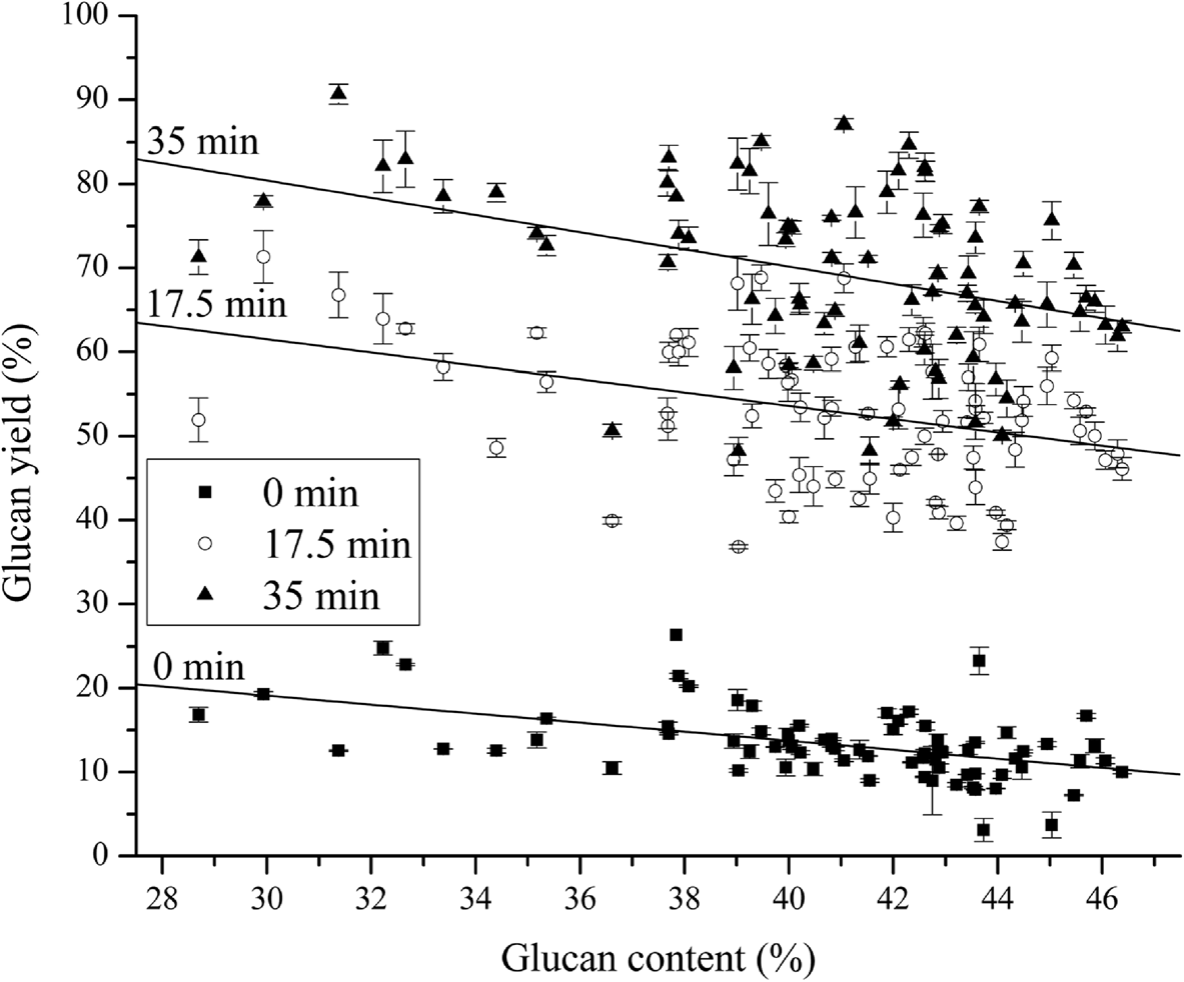
**HTPH glucan yields 80 *Miscanthus*vs. glucan contents in raw *Miscanthus*at 3 different pretreatment times.** HTPH glucan yields on basis of original glucan content in raw *Miscanthus* of 80 *Miscanthus*.

**Figure 4:**
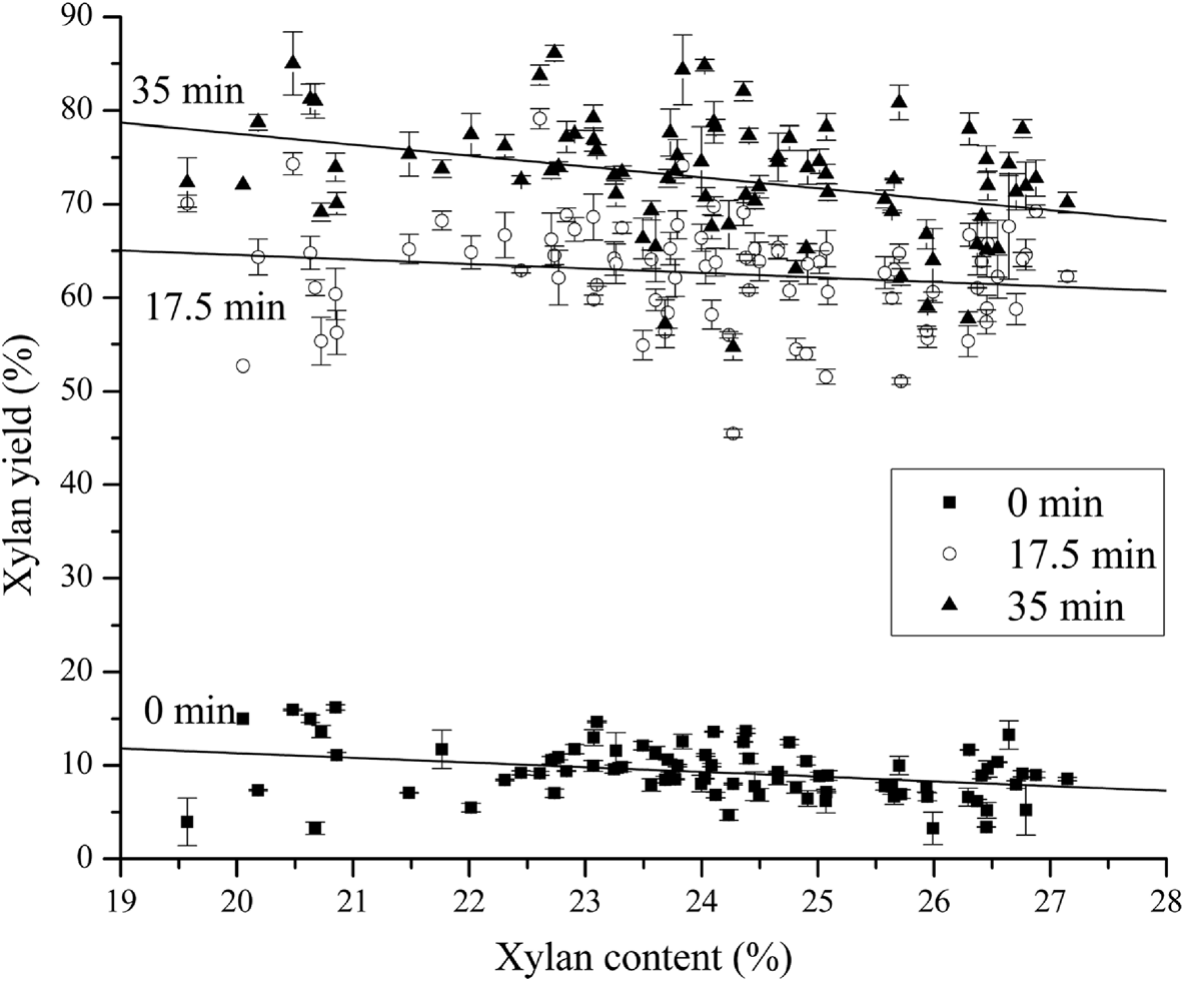
**HTPH Xylan yields of 80 *Miscanthus*vs. glucan contents in raw *Miscanthus*at 3 different pretreatment times.** HTPH xylan yields on basis of original glucan content in raw *Miscanthus* of 80 *Miscanthus*.

Figures 3 and 4 show overall glucan yields (Stage 1+ Stage 2) versus original glucan content and overall xylan yields (Stage 1+ Stage 2) versus original xylan content for each *Miscanthus* sample to further investigate impacts of carbohydrate contents on glucan and xylan yields. The percent theoretical glucan yields based on original glucan content in raw *Miscanthus* (g/g original glucan) in Figure 3 ranged from 3.1% to 26.4% with averages of 13.3% for no pretreatment, from 36.8% to 71.3% with an average of 52.9% for 17.5 min pretreatment, and from 48.6% to 90.6% with an average of 69.3% for 35 min pretreatment. These HTPH results showed that theoretical glucan yields generally decreased as glucan content increased in raw *Miscanthus*. However, glucan mass yields (g/100 g dry weight *Miscanthus*), calculated as a percent of the total dry weight of raw *Miscanthus* tended to increase with glucan content of raw *Miscanthus* (in Additional file 3: Figure S1, glucan yields in column A7 for a 17.5 min pretreatment and in column A9 for a 35 min pretreatment). In other words, as glucan content in raw *Miscanthus* increased, it became more difficult to degrade glucan into glucose through pretreatment and enzymatic hydrolysis by HTPH under the experimental conditions applied even though the quantity of released glucose generally increased due to the greater amount. This negative effect became more significant when the pretreatment time was increased from 0 to 17.5 min and further to 35 min. The mechanism can be a combination of various factors and requires further investigation. A plausible explanation is inhibition of cellulases by higher concentrations of sugars released during HTPH from *Miscanthus* genotypes that contained higher amounts of glucan and/or xylan, especially when higher xylan yields were obtained as the pretreatment time was increased. It was reported recently that not only glucose but also xylose and particularly oligomeric xylan are strong inhibitors of cellulases [41,42]. Another possible mechanism would be greater amounts of cellulose presenting a more structured obstacle to enzyme action. 

The percent of theoretical xylan yields ranged from 3.3% to 16.2% with an average of 9.3% for no pretreatment, from 45.5% to 79.1% with an average of 62.6% for 17.5 min pretreatment, and from 54.7% to 86.1% with an average of 72.9% for 35 min pretreatment (Figure 4). Xylan content in raw *Miscanthus* did not significantly affect xylan yields calculated on basis of the original xylan content of raw *Miscanthus*. Xylan content had more negative effects on theoretical xylan yields for unpretreated *Miscanthus* (0 min) than those for 17.5 min and 35 min pretreatments at 180°C. With hydrothermal pretreatment at 180°C for 17.5 min and 35 min, HTPH results for 80 *Miscanthus* genotypes indicated that pretreatment conditions had a greater impact on xylan hydrolysis than xylan content in raw *Miscanthus* genotypes, in contrast to the findings for glucan yields in Figure 3.

**Figure 5:**
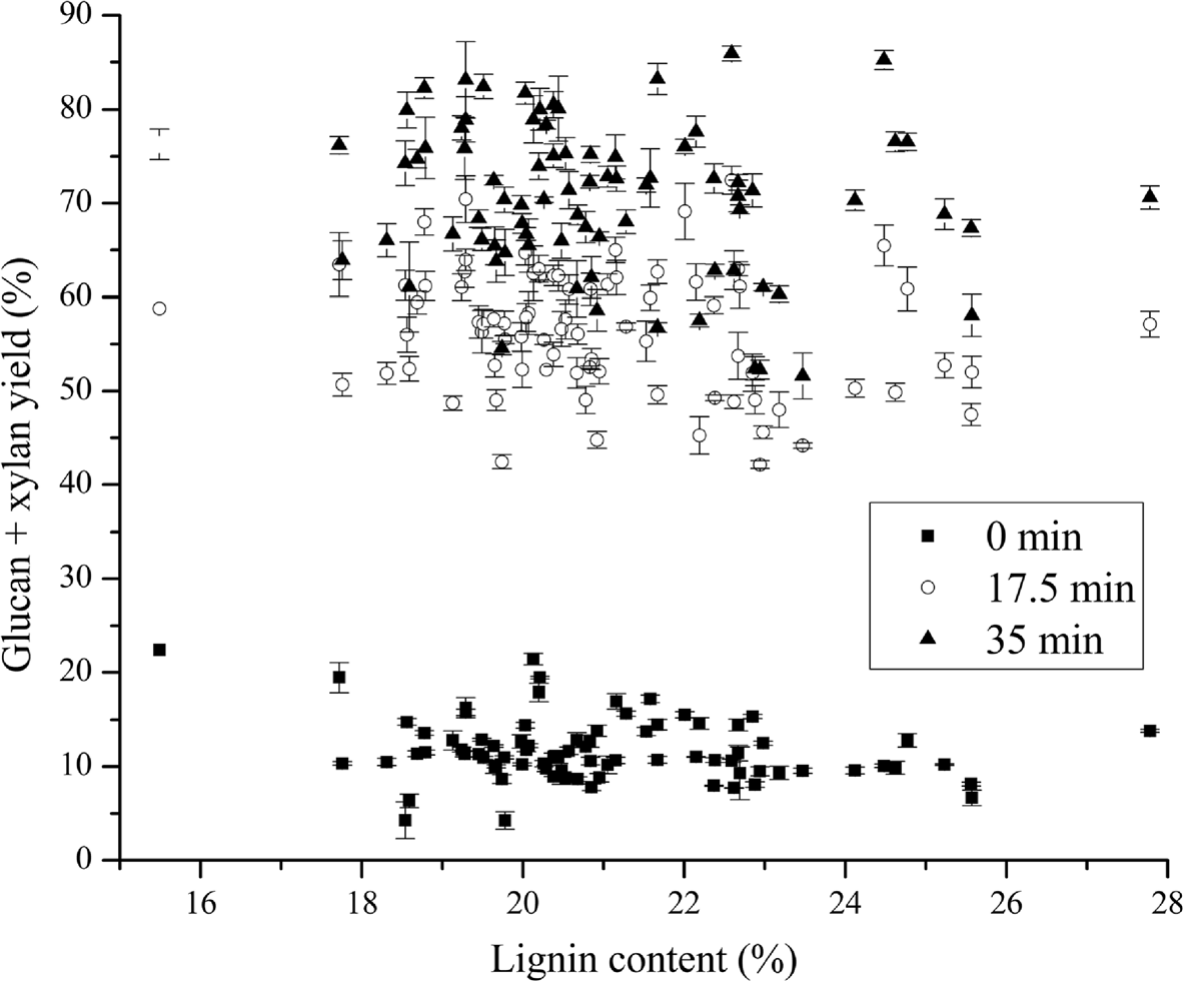
**HTPH glucan and xylan yields of 80 *Miscanthus*samples vs. lignin contents in raw *Miscanthus.*** HTPH glucan and xylan yields on basis of glucan + xylan content in raw *Miscanthus* of 80 *Miscanthus* samples.

The negative impacts of lignin on glucan and xylan mass yields (g/100 g raw *Miscanthus*) are shown in Additional file 3: Figure S1. In order to further investigate the impact of lignin content on overall glucan and xylan yields, the overall percent theoretical glucan and xylan yields based on original glucan and xylan contents are plotted versus lignin content of raw *Miscanthus* for 80 *Miscanthus* samples following hydrothermal pretreatment at 180°C for 0 min, 17.5 min, and 35 min and subsequent enzymatic hydrolysis of the pretreated whole slurry in the HTPH system in Figure 5. The high scatter and significant deviation of glucan and xylan yields from a linear fit to lignin content show that lignin content was not a dominant factor in controlling hydrolysis through hydrothermal pretreatment followed by enzymatic hydrolysis of the whole slurry at a high enzyme loading of 75 mg of cellulase plus 25 mg of xylanase protein/g of total glucan plus xylan in the raw biomass.

Table 3 summarizes the 80 *Miscanthus* genotypes according to sample numbers provided by Mendel Biotechnology showing the highest and lowest sugar yields in terms of both mass yield (g of 100 g dry weight raw *Miscanthus*) and percentage of theoretical yield (g sugar per gram of original glucan and xylan contents in raw *Miscanthus*) for no pretreatment and for a pretreatment time of 35 min as measured by HTPH. Overall, the genotypes with the highest or lowest sugar yields through pretreatment were different from those with the highest or lowest sugar yields without pretreatment. The genotypes showing the highest mass yields were totally different from those with the highest theoretical yields, indicating that genotypes less resistant to HTPH did not necessarily release the highest quantity of sugars. The genotypes with the highest glucan plus xylan yields on the basis of both total mass and percentage of theoretical yields were similar to those with highest glucan yields because of the higher glucan content. The genotypes showing the highest glucan or glucan plus xylan yields without pretreatment contained lignin contents as low as 15.5% and 17.7%. However, the lignin contents of the genotypes realizing the highest glucan or glucan plus xylan yields with pretreatment ranged from 21.7% to 22.6%, slightly higher than the 21.0% average lignin content of all 80 *Miscanthus* genotypes. 112 (*M. sacchariflorus/M. sinensis*) and 229 (*Miscanthus lutarioriparius*) showed similarly high glucan and xylan theoretical yields even though they had much different lignin contents. These results suggest that not only lignin content but also lignin composition impacted sugar yields, similar to results for a study of populus variants [28]. The lowest sugar yields were from two genotypes: 1) Sample 33 (*M. sinensis*) with a glucan plus xylan content of 70.1% had the lowest glucan and glucan plus xylan theoretical yields without pretreatment and 2) Sample 140 (*M. sacchariflorus/M. sinensis*) with a glucan plus xylan content of 60.9% had the lowest glucan, xylan, and glucan plus xylan theoretical yields following pretreatment for 35 min. Thus, these two samples displayed strong recalcitrance even though both glucan and xylan content were close to the average values. In addition, the same genotypes maintained the highest and lowest glucan or xylan theoretical yields as the pretreatment time was extended from 17.5 to 35 min at 180°C, and these varieties were not the same as those showing the highest or lowest theoretical yields without pretreatment. This trend indicated that enzymatic hydrolysis of raw *Miscanthus* without pretreatment would not be effective for rapidly identifying varieties with high yield potential. 

**Table 3:** **Summary of HTPH results for *Miscanthus*genotypes displaying highest and lowest sugar yields **

**Description**	Pretreatment time (min) **	***Miscanthus*sample No.**	**Compositions (g/100 g dw)**			**Sugar yield**		
			**Glu**	**Xyl**	**Lig**	**Glu**	**Xyl**	**Glu + Xyl**
**Highest yields**								
Glu + xyl mass yield*	0	70	43.6	26.6	17.7	10.2	3.5	13.7
	35	112	42.3	25.7	21.7	35.8	20.8	56.6
% of Glu + xyl theoretical yield#	0	4	37.8	20.1	15.5	26.4	15.0	22.4
	35	229	41.1	22.6	22.6	87.1	85.4	86.5
Glu mass yield	0	70	43.6	26.6	17.7	10.2	3.5	13.7
	35	229	41.1	22.6	22.6	35.8	19.3	55.1
% of Glu theoretical yield	0	4	37.8	20.1	15.5	26.4	15.0	22.4
	35	155	29.9	22.7	22.0	77.9	73.6	76.1
Xyl mass yield	0	70	43.6	26.6	17.7	10.2	3.5	13.7
	35	22	42.6	26.8	20.4	34.9	20.9	55.8
% of Xyl theoretical yield	0	152	32.2	20.8	20.1	24.8	16.2	21.4
	35	191	37.7	22.7	19.5	80.1	86.1	82.4
**Lowest yields**								
Glu + xyl mass yield*	0	33	43.7	26.4	19.8	1.4	1.6	3.0
	35	140	36.6	24.3	22.9	18.6	13.3	31.8
% of Glu + xyl theoretical yield#	0	33	43.7	26.4	19.8	3.1	6.2	4.2
	35	141	39.0	23.7	23.5	48.2	57.2	51.6
Glu mass yield	0	33	43.7	26.4	19.8	1.4	1.6	3.0
	35	140	36.6	24.3	22.9	18.6	13.3	31.8
% of Glu theoretical yield	0	33	43.7	26.4	19.8	3.1	6.2	4.2
	35	141	39.0	23.7	23.5	48.2	57.2	51.6
Xyl mass yield	0	259	40.9	20.7	24.1	5.2	0.7	5.9
	35	140	36.6	24.3	22.9	18.6	13.3	31.8
% of Xyl theoretical yield	0	259	40.9	20.7	24.1	12.8	3.3	9.6
	35	140	36.6	24.3	22.9	50.7	54.7	52.3

*mass yield calculated on basis of 100 g dry weight raw *Miscanthus*.

#percent of theoretical yield was calculated on basis of the amount of original glucan or xylan contents in raw *Miscanthus*.

** The highest or lowest yields of HTPH for the pretreatment of 0 or 35 min.

Glu-glucan, xyl-xylan, lig-lignin.

Overall, these 80 genotypes represented similarity of cellulosic biomass samples as well as diversity according to the range of the compositional data and fermentable sugars results. The compositional data for dry *Miscanthus* samples ranged from 27.6% to 48.8% glucan with an average of 40.8% and a standard deviation of 3.8%, from 18.6% to 28.0% xylan with an average of 24.1% and a standard deviation of 2.0%, and from 15.4% to 27.8% K-lignin with an average of 20.9% and a standard deviation of 2.0%. The covariances of glucan and K-lignin, xylan and K-lignin, and glucan and xylan were found to be −2.4, -0.6, and 2.6, respectively. The range of sugar yields also showed that many samples followed a general trend besides some significant outliners. The maximum glucan yields (28.2 g glucan/100 g dry biomass and 35.8 g glucan/100 g dry biomass) were almost two times the minimum glucan yields (14.4 glucan/100 g dry biomass and 18.6 g glucan/100 g dry biomass) for 17.5 min and 35 min pretreatment, respectively. The difference between maximum and minimum xylan yields was smaller than that of glucan for both 17.5 min and 35 min pretreatment. The maximum glucan yields (28.2 g glucan/100 g dry biomass) for 17.5 min pretreatment were similar to the average glucan yield of 80 *Miscanthus* genotypes for 35 min pretreatment. The maximum xylan yields (18.6 g glucan/100 g dry biomass) for 17.5 min pretreatment were greater than the average xylan yield (17.5 g glucan/100 g dry biomass) of 80 *Miscanthus* genotypes for 35 min pretreatment. The covariances of glucan and xylan yields (%) were found above 4.2, 14.0, and 17.7 for 0 min, 17.5 min, and 35 min pretreatment followed by co-hydrolysis, respectively.

Among these 80 *Miscanthus* genotypes, *M. sacchariflorus/M. sinensis* and *Miscanthus lutarioriparius* were the top two in terms of overall sugar theoretical yields of 83.2% and 86.5%, respectively, after hydrothermal pretreatment for 35 min at 180°C followed by enzymatic hydrolysis. These results were equivalent to mass yields of 56.6 g glucan plus xylan/100 g dry *Miscanthus* and 55.1 g glucan plus xylan/100 g dry *Miscanthus*, respectively. Thus, the HTPH system enabled identification of several *Miscanthus* genotypes with potential for high sugar release. On this basis, *M. sacchariflorus/M. sinensis* and *Miscanthus lutarioriparius* were selected for deconstruction in a flowthrough pretreatment to develop more detailed data on the time release patterns of glucan, xylan, and lignin that can help us understand what influences key performance differences. Other samples with greater than 60% overall sugar (glucan + xylan) theoretical yields are of potential interest for more detailed study.

This rapid selection method for fermentable sugar yields could be directly converted to ethanol yields according to the current sugar-to-ethanol conversion yield model/calculation [38,43]. This calculation could be applied to different *Miscanthus* genotypes and other biomass samples and further coupled with the corresponding biomass productivity model under different growing conditions to estimate ethanol yields for cultivation of certain lands with various biomass varieties [9,13]. Therefore, incorporating biomass productivity models with this HTPH screening method would enable better estimation of cellulosic ethanol production to assist in development of new energy crops and testing of cultivation conditions.

## Conclusions

Compositional analysis of 80 *Miscanthus* genotypes showed glucan contents ranging from 28.7% to 46.4%, xylan contents from 19.6% to 27.1%, and total glucan plus xylan contents from 49.6% to 72.0% by dry weight. Thus, *Miscanthus* can have greater carbohydrate content than many other types of fast growing plants and hold promise for high ethanol yields. However, the large variability in composition among *Miscanthus* genotypes shows that appropriate genotypes must be chosen to realize this potential.

Application of our novel high throughput system (HTPH) for hydrothermal pretreatment at 180°C followed by enzymatic hydrolysis of all 80 *Miscanthus* genotypes revealed that *M. sacchariflorus/M. sinensis* and *Miscanthus lutarioriparius* achieved the highest glucan plus xylan mass yields of 56.6 g/100 g and 54.6 g/100 g, respectively, after pretreatment for 35 minutes. The total glucan plus xylan content in *Miscanthus* appeared to influence both mass and theoretical yields, while lignin and ash contents did not have a predictable influence on performance. Because the total glucan plus xylan yields by mild hydrothermal pretreatment and co-hydrolysis of the 80 *Miscanthus* genotypes showed strong correlations to those at more severe (close to optimal) hydrothermal pretreatment and co-hydrolysis conditions, the mild pretreatment results could be used as a faster and lower cost preliminary indicator of promising cellulosic biomass that could realize high yields of fermentable sugar.

## Methods

### Materials

*Miscanthus x giganteus cv. Illinois*, a hybrid of *Miscanthus sacchariflorus* and *Miscanthus sinensis*[44] harvested in Illinois in the fall of 2007, was provided by Mendel Biotechnology, Inc. in Hayward, California. *Miscanthus x giganteus cv. Illinois* materials, including leaves and stalks, were cut to shorter lengths, sealed in heavy duty zipped bags, and stored at −18°C in a laboratory freezer. An appropriate amount of frozen *Miscanthus* was thawed at a temperature no higher than 25°C and mixed completely. The material was then ground with a laboratory mill (model 4, Arthur H. Thomas Company, Philadelphia, PA) to pass between Sieve 20 and Sieve 60 and obtain particles over a size range of 250–850 μm for experiments and analysis. The most cultivated *Miscanthus* genotype in the United States, *Miscanthus x giganteus cv. Illinois*, was used in our hydrothermal pretreatment study to find appropriate conditions to compare other genotypes. Single plants of another 80 *Miscanthus* genotypes (including *Miscanthus sinensis, Miscanthus sacchariflorus*, hybrids of these two, *M.* x *giganteus*, *M. transmorrisonensis*, *M. tinctorius* and *M. lutarioriparius*) were grown in one field plot at Klein-Wanzleben, Germany. Selected plants from collections or from crosses were added annually to the field plot, and biomass was harvested every year before the new shoots appeared in the spring. The plants did not receive fertilizer. Plants sampled for this study were between 2 and 17 years old, grown during the growing season 2007, and harvested by hand in April 2008. *Miscanthus* straw was shredded with a garden shredder and milled with a hammer mill. Plant origin, plant age, and monthly temperature and rainfall for the 2007 growing season are listed in Additional file 1: Table S1 and Additional file 2: Table S2. The 2007 growing season was characterized by higher than average temperature and rainfall. All of these samples were air dried and milled to less than 2 mm prior to shipping in sample bags to the University of California Riverside, where they were stored at −18°C in a laboratory freezer.

### *Miscanthus* compositional analysis

The moisture content of the prepared *Miscanthus* samples was determined with a laboratory moisture analyzer (Mettler Toledo, Model: HB43 Halogen Moisture Analyzer, Columbus, OH). Ash content was analyzed according to NREL Laboratory Analytical Procedures (Technical Report NREL/TP-510-42622) [45] as was extractives content (Technical Report NREL/TP-510-42619) [46]. Klason lignin, glucan, and xylan contents were determined following the modified NREL Laboratory Analytical Procedure (Technical Report NREL/TP-510-42618) [47]. This procedure employed a two-step acid hydrolysis: 1) about 300 mg substrate was placed into a vial and hydrolyzed in 72% (w/w) sulphuric acid at 30°C for 1 hour and 2) the substrate was further hydrolyzed in 4% (w/w) sulphuric acid at 121°C for 1 hour. The sugars in the liquid were determined by HPLC.

### Batch hydrothermal pretreatment in tubular reactors

Tubular reactors (Hastelloy C-276, O.D. 0.0127 m (0.5”) with wall thickness of 0.0008890 m (0.035”), length of 0.1524 m (6”), and volume of 0.0143 L (14.3 ml)) were employed for batch pretreatment of *Miscanthus x giganteus cv. Illinois* to set baseline conditions. These reactors were heated in 4 kW fluidized sand baths (Model SBL-2D, Technical Co., Princeton, NJ), with the internal temperature monitored with a K type thermocouple probe (Omega KQSS-316 G-12, Omega Engineering Co., Stamford, CT). The heat-up time to final reaction temperature was less than 200 seconds and included in the stated reaction time. The heat-up time was slightly longer for the higher temperature than for the lower temperature operation. Cooling down in a water bath to room temperature took about 40 seconds, which was not included in the reaction time.

*Miscanthus x giganteus cv. Illinois* was presoaked in water overnight at a solids loading of 10 wt% for hydrothermal pretreatments at 180°C, 200°C, and 220°C. Following pretreatments, the slurry was separated into a liquid hydrolysate and pretreated solids by vacuum filtration using a 0.22 μm glass fibber filter (09-804-110A, Fisher Science, Pittsburgh, PA). The pretreated solids were washed thoroughly with deionized water before compositional analysis and sequential enzymatic hydrolysis. Sugar yields in the liquid from just hydrothermal pretreatment were designated as Stage 1 sugar yields, and those from subsequent enzymatic hydrolysis of the pretreated solids were labelled as Stage 2 sugar yields.

### High throughput pretreatment and co-hydrolysis (HTPH)

A novel high throughput pretreatment and enzymatic hydrolysis system (HTPH) was used for rapid screening of 80 *Miscanthus* varieties for sugar yields from coupled pretreatment and enzymatic hydrolysis [29]. The method was developed and proved equally effective as conventional batch reactors followed by washed solids hydrolysis [29]. The custom made well-plate consisted of 96 Hastelloy round cups (i.d 6.9 mm x 10.7 mm inside length) with reaction volumes of 300 μL resting on an aluminium bottom plate, covered with a silicone gasket and stainless steel plate, and clamped tightly to contain the contents at pretreatment pressures and temperatures. This assembly was placed horizontally and lengthwise inside a custom made steam chamber made of readily available steam rated (to 1 MPa steam pressure) 316 stainless steel 0.102 m (4”) diameter fittings (McMaster, Santa Fe Springs, CA). A ball valve at one end allowed easy access for loading and unloading. Steam was generated by a high pressure steam boiler (FB-075-L, Fulton Companies, Pulaski, NY) and connected to the chamber along with cooling water. A reaction volume of 250 μL with ~2.6 mg *Miscanthus*(i.e., 1%w/w solids) and 247.4 μL water (8 channel pipetter, 30–300 μL, Eppendorf) was added to each well of the HTPH system. *Miscanthus* was incubated at room temperature for 4 h before pretreatment. *Miscanthus* samples were pretreated in the HTPH system at 180°C for 0, 17.5, and 35 min. After the mixture of the liquid hydrolysate and pretreated solid was cooled down, the plate was opened, and an enzyme loading of 75 mg of cellulase plus 25 mg of xylanase protein/g of total glucan plus xylan for raw *Miscanthus x giganteus cv. Illinois* was achieved by adding 20 μL mixture of 0.625 M citric acid buffer (pH 4.7), 0.125 g/L sodium azide, and enzymes (including 6.08 mg/ml Specyme CP cellulase and 2.03 mg/ml Multifect xylanase) to each well. Such high enzyme loading was applied to overcome possible inhibitory effects of compounds derived from pretreatment under different pretreatment conditions on sugar release. Xylanase was applied to hydrolyze xylooligomers in the liquid hydrolysate for co-hydrolysis, which was different from the enzymatic hydrolysis of pretreated solids in the batch reactor. Following addition of enzymes, sodium azide, and buffer, the plate was re-sealed and placed in an incubation shaker (Multitron Infors-HT, ATR Biotech, Laurel, MD) at 50°C, 150 rpm for 72 h. Samples were filtered by 2 mL centrifuge filter with pore size of 0.20 μm (2 mL centrifuge filter (Catalogue no. 24137), Grace Davison, Deerfield, IL) immediately then frozen for sugar analysis.

### Sugar analysis

Sugar monomers in the liquids from pretreatment and enzymatic hydrolysis were analyzed quantitatively by a Waters HPLC system (model 2695) equipped with a 2414 refractive detector and a Waters 2695 auto sampler using Waters Empower™ 2 software (Waters Co., Milford, MA). Bio-Rad Aminex HPX-87 H and Bio-Rad Aminex HPX-87P columns (Bio-Rad Laboratories, Hercules, CA) were employed for separation of sugars for quantification.

The concentrations of total xylan and glucan in the hydrolysate were determined by post-hydrolysis with 4% w/w sulphuric acid at 121°C for 1 hour according to NREL Laboratory Analytical Procedure (*Technical Report* NREL/TP-510-42623) [48]. Both glucan and xylan yields in Stage 1 were reported as the sum of monomer and oligomer yields.

### Enzymatic hydrolysis

Washed solids from hydrothermal pretreatment of *Miscanthus* in the batch tubes were enzymatically hydrolyzed at 2% solids loadings with a pH value of 4.8 at 50°C in duplicates by following modified NREL Laboratory Analytical Procedure (Technical Report NREL/TP-510-42629) [49] using Spezyme CP (62 FPU/ml, protein content 116.0 mg/ml, Genencor, Rochester, NY) and Novozymes 188 (β-glucosidase, activity 665.0 CBU/ml, protein content 125.0 mg/ml, Franklinton, NC). The ratio of cellulase filter paper activity to beta-glucosidase activity was FPU: CBU = 1:4, and the total enzyme loading was 60 FPU/g (glucan + xylan) in the pretreated solids. Hydrolysis samples were collected at 72 hours, and sugar concentrations were determined for calculation of Stage 2 glucan and xylan yields.

### Calculations

The log of the severity parameter (log*R*_*0*_) for hydrothermal pretreatment was defined as a function of pretreatment temperature T(°C) and pretreatment time t(min), as [39]:

(1) $${\text{R}}_{0}=\text{t}\cdot \text{exp}\left(\frac{\text{T}-100}{14.75}\right)$$

Glucan and xylan yields and overall glucan and xylan yields for batch pretreatment and enzymatic hydrolysis were calculated as:

(2) $$\text{glucan}\;\text{yield(\%})=\frac{\text{glucose}\;conc.\;\text{in the liquid hydrolysate}\;\left(g/L\right)*total\;volume\;(\text{L})*0.9}{\text{initial}\;Miscanthus\;amount\;\left(g\right)*original\;\text{glucan}\;content\;\text{in t}he\;Miscanthus(\%)}\times 100$$

(3) $$xylan\;yield\left(\%\right)=\frac{\text{x}ylose\;conc.\text{in the liquid hydrolysate}\;\left(g/L\right)*total\;volume(\text{L})*0.8801}{\text{initial}\;\;\text{solid}\;Miscanthus\left(g\right)*original\;\text{x}yl\text{an}\;content\;\text{in t}he\;Miscanthus(\%)}\times 100$$

(4) $$\text{glucan}+\text{xylan}\;\text{yield}(\%)=\frac{\text{glucan yield}*\text{initial glucan in}\;\text{Miscanthus}(g)+\text{xylan yield}*\text{initial xylan in}\;\text{Miscanthus}(g)}{\text{initial glucan}+\text{xylan in}\;\text{Miscanthus}(g)}\times 100$$

These equations were applied to determine yields in Stage 1 (pretreatment) and Stage 2 (enzymatic hydrolysis). For Stage 1, sugar yields included both monomer and oligomers determined through post hydrolysis of the liquid hydrolysate. Sugar yields were calculated as percent of the theoretical maximum on the basis of original glucan and/or xylan content in raw *Miscanthus* unless otherwise specified. Overall glucan plus xylan yields were defined as the sum of glucan plus xylan yields from Stage 1 and Stage 2.

The calculations for the high throughput pretreatment and co-hydrolysis were based on the sugar amounts after co-hydrolysis following a previously published method [29]. Monomeric sugars in the liquid hydrolysate were measured after pretreatment and co-hydrolysis, and sugar yields were calculated by equations 2–4.

## Abbreviations

HTPH: High throughput pretreatment and hydrolysis; FPU: Filter paper unit; CBU: Cellobiase unit.

## Competing interests

CEW is cofounder of Mascoma Corporation and chair of their Scientific Advisory Board. CEW is also member of the Scientific Advisory Board of Mendel Biotechnology, Inc. CEW is also founding Editor in Chief of this Journal BfB.

The other authors declare that they have no competing interests other than support of this research by Mendel Biotechnology.

## Authors’ contributions

TZ carried out this study under the supervision of BY and CEW. KJ provides logistic and background information for all *Miscanthus* samples. All the authors read and accepted this final manuscript.

## Supplementary Material

Additional file 1 **Table S1.** Information of the Miscanthus straw samples from Mendel Biotechnology, Inc. and composition data from UCR Summary of Miscanthus pretreatment data reported in the literature. Click here for file

Additional file 2 **Table S2.** Weather data from airport Magdeburg, near Klein-Wanzleben. Click here for file

Additional file 3 **Figure S1.** Normalized scatter matrix of 80 *Miscanthus* compositions, HTPH glucan and xylan mass yields (g/100 g dw raw *Miscanthus*). HTPH glucan and xylan mass yields (g/100 g dw raw *Miscanthus*) from hydrothermal pretreatment at 180°C in 0, 17.5 min, and 35 min. Click here for file
