# Supplementary material for: Rapid selection and identification of Miscanthus genotypes with enhanced glucan and xylan yields from hydrothermal pretreatment followed by enzymatic hydrolysis
Source: Biotechnol Biofuels. 2012 Aug 3;5:56. doi: 10.1186/1754-6834-5-56 (PMC3494522; doi:10.1186/1754-6834-5-56)
Supplement: Additional file 1 — Table S1. Information of the Miscanthus straw samples from Mendel Biotechnology, Inc. and composition data from UCR Summary of Miscanthus pretreatment data reported in the literature. [file 1754-6834-5-56-S1.docx]

| Supplement Table 1. Information of the *Miscanthus* straw samples from Mendel Biotechnology, Inc. and composition data from UCR | | | | | | | | | | | | |
| --- | --- | --- | --- | --- | --- | --- | --- | --- | --- | --- | --- | --- |
| List of straw samples from Tinplant harvest 2008 from Mendel Biotechnology, Inc. | | | | | Composition data from UCR (based on dry weight) | | | | | | | |
| Sample # | Genotype | Age * | planted | plant origin | k-lignin (%) | | Glucan (%) | | Xylan (%) | | Ash (%) | |
|  |  |  |  |  | Aver. | stdev | Aver. | stdev | Aver. | stdev | Aver | stdev |
| 4 | Miscanthus transmorrisonensis | 17 | 1991 | collected/obtained from: Germany | 15.5 | 0.03 | 37.9 | 0.23 | 20.1 | 0.69 | 7.4 | 0.09 |
| 5 | Miscanthus sinensis, Grosse Fontaene | 17 | 1991 | collected/obtained from: Germany | 18.8 | 0.28 | 39.6 | 0.53 | 24.7 | 0.41 | 3.6 | 0.06 |
| 6 | Miscanthus sinensis Goliath | 17 | 1991 | collected/obtained from: Germany | 18.7 | 0.08 | 40.1 | 0.50 | 25.0 | 0.36 | 3.1 | 0.05 |
| 10 | Miscanthus xgiganteus | 17 | 1991 | collected/obtained from: Denmark | 22.6 | 0.27 | 44.0 | 0.62 | 21.5 | 0.30 | 1.9 | 0.05 |
| 14 | Miscanthus sinensis, Silberfeder | 16 | 1992 | collected/obtained from: UK | 17.8 | 0.32 | 48.6 | 0.25 | 24.2 | 1.05 | 3.6 | 0.06 |
| 17 | Miscanthus sinensis, Grosse Fontaene | 16 | 1992 | collected/obtained from: Germany | 19.5 | 0.08 | 40.2 | 0.34 | 23.3 | 0.20 | 4.1 | 0.17 |
| 18 | Miscanthus xgiganteus | 17 | 1991 | collected/obtained from: Denmark | 25.6 | 1.18 | 43.6 | 0.47 | 19.6 | 0.51 | 2.2 | 0.07 |
| 19 | Miscanthus sacchariflorus | 16 | 1992 | collected/obtained from: Germany | 18.5 | 0.38 | 45.0 | 0.88 | 26.8 | 0.82 | 2.4 | 0.00 |
| 21 | Miscanthus sinensis/M. sacchariflorus | 15 | 1993 | F1 hybrid of polycross M. sinensis x M. sacchariflorus | 20.4 | 0.05 | 43.0 | 2.05 | 26.5 | 0.88 | 3.1 | 0.06 |
| 22 | Miscanthus sinensis/M. sacchariflorus | 15 | 1993 | F1 hybrid of polycross M. sinensis x M. sacchariflorus | 20.4 | 0.28 | 42.6 | 0.70 | 26.8 | 0.36 | 3.4 | 0.05 |
| 23 | Miscanthus sinensis/M. sacchariflorus | 15 | 1993 | F1 hybrid of polycross M. sinensis x M. sacchariflorus | 18.3 | 0.73 | 46.1 | 0.73 | 25.1 | 0.76 | 3.4 | 0.09 |
| 25 | Miscanthus sinensis | 15 | 1993 | F1 hybrid of polycoss M. sinensis | 19.5 | 0.25 | 40.7 | 0.30 | 24.0 | 0.83 | 3.4 | 0.09 |
| 27 | Miscanthus sinensis | 15 | 1993 | F1 hybrid of polycoss M. sinensis | 20.7 | 0.34 | 38.9 | 0.35 | 23.6 | 0.47 | 3.7 | 0.11 |
| 32 | Miscanthus sinensis/M. sacchariflorus | 15 | 1993 | F1 hybrid of polycross M. sinensis x M. sacchariflorus | 19.3 | 0.13 | 41.3 | 0.09 | 24.0 | 0.49 | 3.3 | 0.06 |
| 33 | Miscanthus sinensis | 15 | 1993 | collected/obtained from: Japan | 19.8 | 0.29 | 43.7 | 0.35 | 26.4 | 0.67 | 3.2 | 0.07 |
| 34 | Miscanthus sinensis/M. sacchariflorus | 15 | 1993 | F1 hybrid of polycross M. sinensis x M. sacchariflorus | 21.2 | 0.34 | 42.6 | 0.72 | 26.9 | 0.71 | 2.5 | 0.04 |
| 36 | Miscanthus sinensis/M. sacchariflorus | 15 | 1993 | F1 hybrid of polycross M. sinensis x M. sacchariflorus | 20.1 | 0.53 | 44.9 | 0.85 | 26.6 | 0.60 | 3.0 | 0.07 |
| 62 | Miscanthus sinensis | 16 | 1992 | F1 hybrid of single pair cross M. sinensis | 21.0 | 0.63 | 46.4 | 1.38 | 25.7 | 1.07 | 2.2 | 0.05 |
| 69 | Miscanthus sinensis | 15 | 1993 | collected/obtained from: Japan | 20.9 | 0.55 | 42.6 | 0.27 | 26.5 | 0.36 | 2.1 | 0.08 |
| 70 | Miscanthus tinctorius | 13 | 1995 | Not known | 17.7 | 0.65 | 43.7 | 1.02 | 26.6 | 0.59 | 2.3 | 0.03 |
| 104 | M. sacchariflorus/M. sinensis | 2 | 2006 | F1 hybrid of M. sacchariflorus x M. sinensis | 20.8 | 0.32 | 40.8 | 0.48 | 24.9 | 0.58 | 3.3 | 0.01 |
| 105 | M. sacchariflorus/M. sinensis | 2 | 2006 | F1 hybrid of M. sacchariflorus x M. sinensis | 20.0 | 0.88 | 42.6 | 0.36 | 24.4 | 0.23 | 2.7 | 0.04 |
| 106 | M. sacchariflorus/M. sinensis | 2 | 2006 | F1 hybrid of M. sacchariflorus x M. sinensis | 20.4 | 0.70 | 39.3 | 0.23 | 23.7 | 0.61 | 3.7 | 0.06 |
| 107 | M. sacchariflorus/M. sinensis | 2 | 2006 | F1 hybrid of M. sacchariflorus x M. sinensis | 20.0 | 0.49 | 45.9 | 1.45 | 22.9 | 0.26 | 3.1 | 0.06 |
| 108 | M. sacchariflorus/M. sinensis | 2 | 2006 | F1 hybrid of M. sacchariflorus x M. sinensis | 19.3 | 0.33 | 41.9 | 0.24 | 24.1 | 0.25 | 3.3 | 0.04 |
| 109 | M. sacchariflorus/M. sinensis | 2 | 2006 | F1 hybrid of M. sacchariflorus x M. sinensis | 18.6 | 0.38 | 42.1 | 1.08 | 24.8 | 0.41 | 3.2 | 0.08 |
| 110 | M. sacchariflorus/M. sinensis | 2 | 2006 | F1 hybrid of M. sacchariflorus x M. sinensis | 21.1 | 0.94 | 39.9 | 0.80 | 26.5 | 0.99 | 2.7 | 0.05 |
| 111 | M. sacchariflorus/M. sinensis | 2 | 2006 | F1 hybrid of M. sacchariflorus x M. sinensis | 20.3 | 0.14 | 41.5 | 0.57 | 25.6 | 0.28 | 3.3 | 0.11 |
| 112 | M. sacchariflorus/M. sinensis | 2 | 2006 | F1 hybrid of M. sacchariflorus x M. sinensis | 21.7 | 0.73 | 42.3 | 0.26 | 25.7 | 0.30 | 3.5 | 0.03 |
| 113 | M. sacchariflorus/M. sinensis | 2 | 2006 | F1 hybrid of M. sacchariflorus x M. sinensis | 19.7 | 0.01 | 42.9 | 0.64 | 22.0 | 0.32 | 2.3 | 0.10 |
| 114 | M. sacchariflorus/M. sinensis | 2 | 2006 | F1 hybrid of M. sacchariflorus x M. sinensis | 20.6 | 0.83 | 43.4 | 1.31 | 23.8 | 0.73 | 3.3 | 0.08 |
| 115 | M. sacchariflorus/M. sinensis | 2 | 2006 | F1 hybrid of M. sacchariflorus x M. sinensis | 19.3 | 0.10 | 39.0 | 0.24 | 23.8 | 0.27 | 2.7 | 0.06 |
| 133 | Miscanthus sinensis | 15 | 1993 | collected/obtained from: Japan | 18.6 | 0.65 | 43.5 | 1.71 | 26.0 | 0.63 | 2.0 | 0.09 |
| 137 | M. sacchariflorus/M. sinensis | 8 | 2000 | F1 hybrid of M. sacchariflorus x M. sinensis | 22.7 | 0.99 | 35.2 | 0.21 | 23.6 | 0.08 | 1.7 | 0.06 |
| 138 | M. sacchariflorus/M. sinensis | 8 | 2000 | F1 hybrid of M. sacchariflorus x M. sinensis | 23.2 | 0.05 | 40.5 | 1.52 | 24.8 | 1.15 | 2.7 | 0.02 |
| 139 | M. sacchariflorus/M. sinensis | 8 | 2000 | F1 hybrid of M. sacchariflorus x M. sinensis | 22.9 | 0.21 | 41.6 | 0.88 | 25.9 | 0.43 | 2.5 | 0.05 |
| 140 | M. sacchariflorus/M. sinensis | 8 | 2000 | F1 hybrid of M. sacchariflorus x M. sinensis | 22.9 | 0.37 | 36.6 | 0.66 | 24.3 | 0.53 | 2.4 | 0.03 |
| 141 | M. sacchariflorus/M. sinensis | 8 | 2000 | F1 hybrid of M. sacchariflorus x M. sinensis | 23.5 | 0.36 | 39.0 | 0.74 | 23.7 | 0.54 | 2.4 | 0.04 |
| 142 | M. sacchariflorus/M. sinensis | 8 | 2000 | F1 hybrid of M. sacchariflorus x M. sinensis | 23.0 | 0.49 | 40.0 | 1.38 | 24.9 | 0.74 | 2.9 | 0.01 |
| 147 | Miscanthus sinensis | 9 | 1999 | F1 hybrid of polycross M. sinensis | 22.4 | 0.31 | 42.8 | 0.68 | 22.5 | 0.31 | 2.1 | 0.06 |
| 148 | Miscanthus sinensis | 9 | 1999 | F1 hybrid of polycross M. sinensis | 22.2 | 0.13 | 42.0 | 0.35 | 20.7 | 0.20 | 2.9 | 0.04 |
| 149 | Miscanthus sinensis | 9 | 1999 | F1 hybrid of polycross M. sinensis | 22.7 | 0.24 | 28.7 | 1.44 | 20.9 | 0.16 | 2.5 | 0.09 |
| 150 | Miscanthus sinensis | 9 | 1999 | F1 hybrid of polycross M. sinensis | 20.2 | 1.00 | 27.7 | 0.40 | 23.1 | 0.78 | 3.5 | 0.03 |
| 152 | Miscanthus sinensis | 9 | 1999 | F1 hybrid of polycross M. sinensis | 20.1 | 0.74 | 32.2 | 0.89 | 20.9 | 0.48 | 3.3 | 0.10 |
| 155 | Miscanthus sinensis | 9 | 1999 | F1 hybrid of polycross M. sinensis | 22.0 | 0.60 | 29.9 | 0.46 | 22.7 | 0.93 | 2.5 | 0.09 |
| 157 | Miscanthus sinensis | 9 | 1999 | F1 hybrid of polycross M. sinensis | 24.6 | 0.63 | 34.4 | 0.60 | 25.1 | 0.35 | 2.6 | 0.05 |
| 160 | Miscanthus sinensis | 9 | 1999 | F1 hybrid of polycross M. sinensis | 27.8 | 0.19 | 35.4 | 1.31 | 24.1 | 0.84 | 2.3 | 0.07 |
| 169 | Miscanthus sinensis | 9 | 1999 | F1 hybrid of polycross M. sinensis | 22.2 | 0.49 | 33.4 | 1.03 | 22.3 | 0.21 | 2.2 | 0.04 |
| 170 | Miscanthus sinensis | 9 | 1999 | F1 hybrid of polycross M. sinensis | 20.9 | 0.70 | 44.2 | 0.31 | 23.5 | 0.24 | 3.1 | 0.04 |
| 172 | Miscanthus sinensis | 9 | 1999 | F1 hybrid of polycross M. sinensis | 21.6 | 0.63 | 39.3 | 2.16 | 20.5 | 0.96 | 2.7 | 0.04 |
| 173 | Miscanthus sinensis | 9 | 1999 | F1 hybrid of polycross M. sinensis | 20.8 | 0.40 | 42.9 | 0.77 | 24.4 | 0.65 | 3.5 | 0.04 |
| 176 | Miscanthus sinensis | 9 | 1999 | F1 hybrid of polycross M. sinensis | 24.5 | 0.85 | 31.4 | 1.05 | 24.1 | 0.60 | 2.7 | 0.06 |
| 178 | Miscanthus sinensis | 9 | 1999 | F1 hybrid of polycross M. sinensis | 22.9 | 0.66 | 40.2 | 0.34 | 20.6 | 0.63 | 3.0 | 0.03 |
| 182 | Miscanthus sinensis | 9 | 1999 | F1 hybrid of polycross M. sinensis | 20.5 | 0.48 | 43.6 | 0.04 | 25.1 | 0.19 | 1.9 | 0.06 |
| 186 | Miscanthus sinensis | 9 | 1999 | F1 hybrid of polycross M. sinensis | 20.8 | 0.48 | 39.7 | 0.62 | 23.7 | 0.38 | 3.0 | 0.02 |
| 187 | Miscanthus sinensis | 9 | 1999 | F1 hybrid of polycross M. sinensis | 25.2 | 0.49 | 42.4 | 1.06 | 23.8 | 0.58 | 2.4 | 0.02 |
| 188 | Miscanthus sinensis | 9 | 1999 | F1 hybrid of polycross M. sinensis | 19.7 | 0.86 | 44.1 | 0.25 | 25.7 | 0.05 | 2.6 | 0.07 |
| 189 | Miscanthus sinensis | 9 | 1999 | F1 hybrid of polycross M. sinensis | 19.1 | 0.16 | 41.4 | 0.42 | 23.1 | 0.26 | 3.1 | 0.10 |
| 190 | Miscanthus sinensis | 9 | 1999 | F1 hybrid of polycross M. sinensis | 19.5 | 0.11 | 37.7 | 0.90 | 22.7 | 0.77 | 2.7 | 0.02 |
| 191 | Miscanthus sinensis | 9 | 1999 | F1 hybrid of polycross M. sinensis | 20.3 | 0.79 | 42.9 | 0.64 | 24.0 | 0.27 | 2.5 | 0.05 |
| 192 | Miscanthus sinensis/M. sacchariflorus | 8 | 2000 | F1 hybrid of polycross M. sinensis x M. sacchariflorus | 20.7 | 0.26 | 43.4 | 1.18 | 24.5 | 0.37 | 2.3 | 0.01 |
| 193 | Miscanthus sinensis/M. sacchariflorus | 8 | 2000 | F1 hybrid of polycross M. sinensis x M. sacchariflorus | 20.5 | 0.24 | 44.5 | 1.52 | 24.5 | 0.78 | 2.1 | 0.01 |
| 194 | Miscanthus sinensis/M. sacchariflorus | 7 | 2001 | F1 hybrid of polycross M. sinensis x M. sacchariflorus | 22.7 | 0.83 | 42.8 | 0.40 | 23.3 | 0.22 | 2.7 | 0.08 |
| 195 | Miscanthus sinensis/M. sacchariflorus | 7 | 2001 | F1 hybrid of polycross M. sinensis x M. sacchariflorus | 21.5 | 0.56 | 37.7 | 0.21 | 22.8 | 0.18 | 3.4 | 0.02 |
| 196 | Miscanthus sinensis | 8 | 2000 | M. sinensis hybrid: Denmark | 25.6 | 0.60 | 43.2 | 0.27 | 20.2 | 0.49 | 1.1 | 0.04 |
| 204 | Miscanthus xgiganteus | 12 | 1996 | collected/obtained from: Denmark | 18.8 | 0.57 | 34.5 | 1.80 | 26.3 | 1.24 | 2.1 | 0.08 |
| 205 | Miscanthus sacchariflorus robustus | 10 | 1998 | collected/obtained from: Germany | 19.2 | 0.15 | 37.7 | 0.75 | 25.6 | 0.37 | 3.6 | 0.15 |
| 208 | M. sacchariflorus/M. sinensis | 3 | 2005 | F1 hybrid of M. sacchariflorus x M. sinensis | 20.0 | 0.07 | 44.3 | 0.91 | 26.7 | 0.63 | 2.4 | 0.03 |
| 209 | M. sacchariflorus/M. sinensis | 3 | 2005 | F1 hybrid of M. sacchariflorus x M. sinensis | 19.7 | 0.26 | 45.6 | 0.03 | 25.9 | 0.37 | 2.7 | 0.07 |
| 210 | M. sacchariflorus/M. sinensis | 3 | 2005 | F1 hybrid of M. sacchariflorus x M. sinensis | 19.8 | 0.38 | 44.5 | 0.39 | 27.2 | 0.25 | 2.9 | 0.04 |
| 211 | M. sacchariflorus/M. sinensis | 3 | 2005 | F1 hybrid of M. sacchariflorus x M. sinensis | 24.8 | 0.46 | 40.0 | 1.22 | 23.1 | 0.64 | 2.3 | 0.08 |
| 212 | M. sacchariflorus/M. sinensis | 3 | 2005 | F1 hybrid of M. sacchariflorus x M. sinensis | 20.0 | 0.29 | 43.6 | 0.97 | 26.4 | 0.42 | 3.1 | 0.08 |
| 213 | Miscanthus tinctorius | 13 | 1995 | Not known | 20.2 | 0.84 | 37.9 | 0.39 | 21.8 | 0.46 | 3.4 | 0.06 |
| 220 | Miscanthus lutarioriparius | 15 | 1993 | collected/obtained from: China | 21.7 | 0.24 | 42.1 | 0.77 | 26.3 | 0.40 | 3.0 | 0.06 |
| 224 | Miscanthus sacchariflorus | 15 | 1993 | collected/obtained from: Japan | 22.6 | 0.49 | 41.1 | 0.47 | 22.6 | 0.36 | 1.1 | 0.01 |
| 229 | Miscanthus lutarioriparius | 11 | 1997 | collected/obtained from: not known | 21.3 | 0.45 | 45.7 | 1.14 | 24.4 | 0.62 | 2.2 | 0.04 |
| 234 | Miscanthus lutarioriparius | 10 | 1998 | collected/obtained from: China | 22.4 | 0.70 | 45.5 | 0.58 | 22.8 | 0.39 | 1.9 | 0.08 |
| 235 | Miscanthus lutarioriparius | 10 | 1998 | collected/obtained from: Japan | 21.2 | 0.35 | 38.1 | 0.31 | 23.3 | 0.39 | 3.2 | 0.09 |
| 243 | Miscanthus sacchariflorus | 15 | 1993 | collected/obtained from: China | 19.6 | 0.31 | 40.8 | 0.19 | 24.7 | 0.46 | 2.4 | 0.01 |
| 257 | Miscanthus tinctorius | 13 | 1995 | Not known | 24.1 | 0.22 | 40.9 | 0.71 | 20.7 | 0.94 | 3.8 | 0.09 |
| 259 | Miscanthus xgiganteus Illinois |  |  | 'Illinois' aliquot of UCR sample originally from Damian | 15.5 | 0.03 | 37.9 | 0.23 | 20.1 | 0.69 | 7.4 | 0.09 |

*Age of the plant in the field at harvest in April 2008
