# Supplementary material for: Rapid selection and identification of Miscanthus genotypes with enhanced glucan and xylan yields from hydrothermal pretreatment followed by enzymatic hydrolysis
Source: Biotechnol Biofuels. 2012 Aug 3;5:56. doi: 10.1186/1754-6834-5-56 (PMC3494522; doi:10.1186/1754-6834-5-56)
Supplement: Additional file 2 — Table S2. Weather data from airport Magdeburg, near Klein-Wanzleben. [file 1754-6834-5-56-S2.docx]

Supplement Table 2. Weather data from airport Magdeburg, near Klein-Wanzleben

| time | temperature [C] | | rainfall [mm] | | sunshine hours | |
| --- | --- | --- | --- | --- | --- | --- |
|  | average | deviation from mean | sum | deviation from mean | sum | deviation from mean |
| 2007 / 01 | 5,9 | +6,3 | 66,8 | 202% | 47,4 | 101% |
| 2007 / 02 | 4,5 | +4,0 | 37,5 | 121% | 59,4 | 85% |
| 2007 / 03 | 7,6 | +3,7 | 49,2 | 129% | 171,4 | 146% |
| 2007 / 04 | 11,9 | +3,9 | 5,2 | 13% | 263,4 | 166% |
| 2007 / 05 | 15,3 | +2,3 | 90,8 | 193% | 229,3 | 106% |
| 2007 / 06 | 18,8 | +2,6 | 130,7 | 211% | 204,5 | 94% |
| 2007 / 07 | 18,8 | +1,3 | 82,1 | 171% | 189,4 | 87% |
| 2007 / 08 | 18,2 | +0,9 | 86,7 | 170% | 210,1 | 101% |
| 2007 / 09 | 14,2 | +0,4 | 103,8 | 288% | 143,3 | 95% |
| 2007 / 10 | 9.0 | -0,5 | 19,2 | 66% | 108,2 | 101% |
| 2007 / 11 | 4,9 | +0,4 | 52,6 | 138% | 41,5 | 74% |
| 2007 / 12 | 2,5 | +1,3 | 20,8 | 51% | 49,1 | 120% |
| Overall 2007 | 11 | +2,2 | 745,4 | 151% | 1717 | 107% |
| 2008 / 01 | 4,7 | +5,1 | 67,3 | 204% | 60,6 | 130% |
| 2008 / 02 | 4,9 | +4,4 | 15.0 | 48% | 114,7 | 165% |
| 2008 / 03 | 5,2 | +1,3 | 41,3 | 109% | 125,3 | 107% |
| 2008 / 04 | 8,8 | +0,8 | 73,1 | 183% | 136.0 | 85% |
| 2008 / 05 | 15,5 | +2,5 | 3,1 | 7% | 289,2 | 134% |
| 2008 / 06 | 18,1 | +1,9 | 42,1 | 68% | 309,2 | 141% |
| 2008 / 07 | 19,3 | +1,8 | 81,8 | 170% | 221,6 | 101% |
| 2008 / 08 | 18,7 | +1,4 | 45.0 | 88% | 186,8 | 90% |
| 2008 / 09 | 13,7 | -0,1 | 44.0 | 122% | 132,1 | 87% |
| 2008 / 10 | 10,2 | +0,7 | 38,2 | 132% | 111,6 | 104% |
| 2008 / 11 | 6.0 | +1,5 | 15,9 | 42% | 34,3 | 61% |
| 2008 / 12 | 1,9 | +0,7 | 26,5 | 65% | 41,8 | 102% |
| Overall 2008 | 10,6 | +1,8 | 493,3 | 100% | 1763,2 | 110% |
