# Supplementary figures and images for: Rapid selection and identification of Miscanthus genotypes with enhanced glucan and xylan yields from hydrothermal pretreatment followed by enzymatic hydrolysis
Source: Biotechnol Biofuels. 2012 Aug 3;5:56. doi: 10.1186/1754-6834-5-56 (PMC3494522; doi:10.1186/1754-6834-5-56)

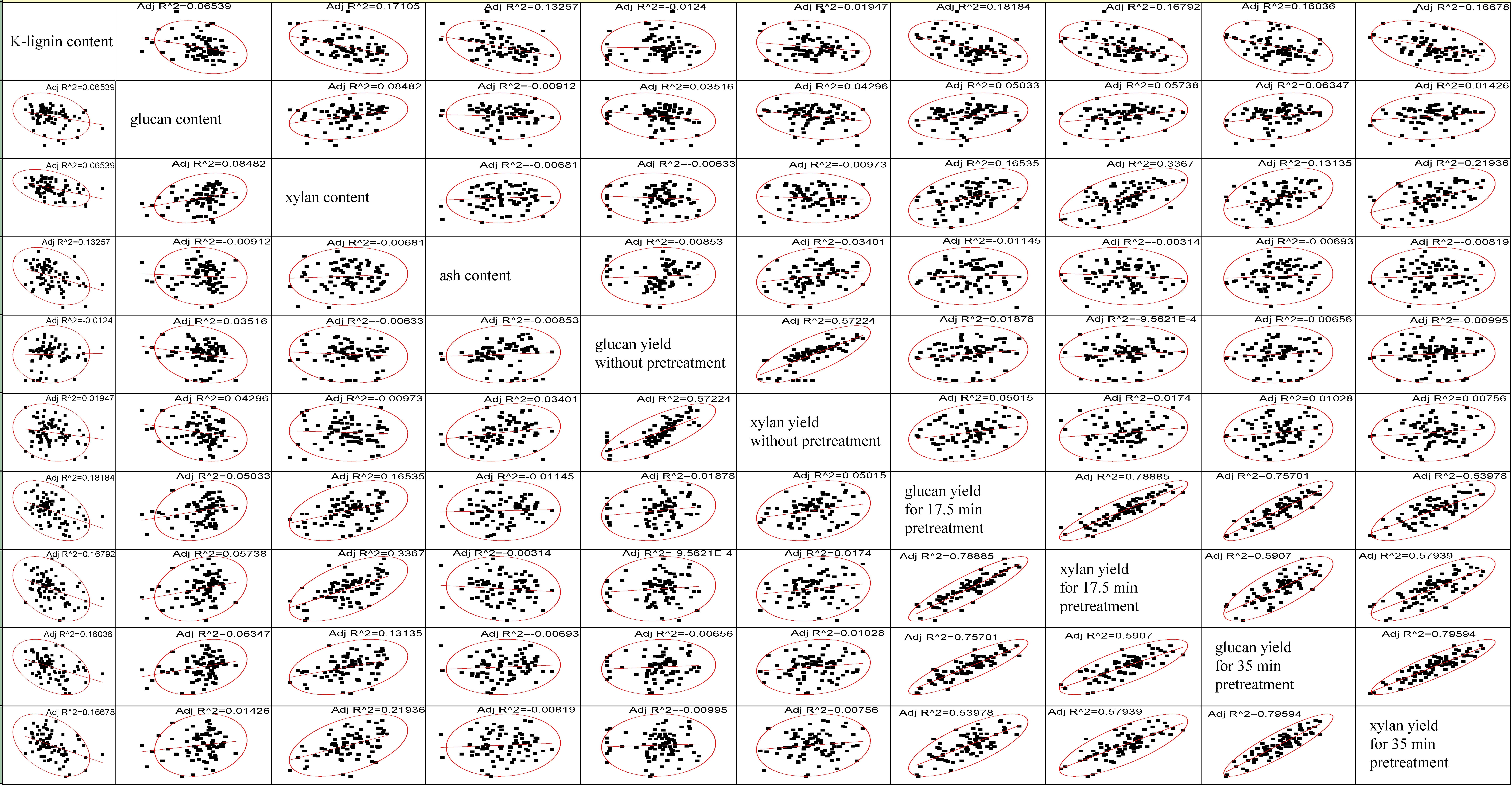

Supplement: Additional file 3 — Figure S1. Normalized scatter matrix of 80 Miscanthus compositions, HTPH glucan and xylan mass yields (g/100 g dw raw Miscanthus). HTPH glucan and xylan mass yields (g/100 g dw raw Miscanthus) from hydrothermal pretreatment at 180°C in 0, 17.5 min, and 35 min. [file 1754-6834-5-56-S3.jpeg]
